# Olfactory Bulb D_2_/D_3_ Receptor Availability after Intrastriatal Botulinum Neurotoxin-A Injection in a Unilateral 6-OHDA Rat Model of Parkinson’s Disease

**DOI:** 10.3390/toxins14020094

**Published:** 2022-01-25

**Authors:** Teresa Alberts, Veronica Antipova, Carsten Holzmann, Alexander Hawlitschka, Oliver Schmitt, Jens Kurth, Jan Stenzel, Tobias Lindner, Bernd J. Krause, Andreas Wree, Martin Witt

**Affiliations:** 1 Department of Anatomy, Rostock University Medical Center, D-18057 Rostock, Germany; teresa.alberts@icloud.com (T.A.); veronica.antipova@medunigraz.at (V.A.); alexander.hawlitschka@med.uni-rostock.de (A.H.); oliver.schmitt@med.uni-rostock.de (O.S.); andreas.wree@med.uni-rostock.de (A.W.); 2 Gottfried Schatz Research Center for Cell Signaling, Metabolism and Aging, Macroscopic and Clinical Anatomy, Medical University of Graz, A-8010 Graz, Austria; 3 Department of Medical Genetics, Rostock University Medical Center, D-18057 Rostock, Germany; carsten.holzmann@med.uni-rostock.de; 4 Center of Transdisciplinary Neuroscience Rostock, D-18147 Rostock, Germany; bernd.krause@med.uni-rostock.de; 5 Department of Nuclear Medicine, Rostock University Medical Center, D-18057 Rostock, Germany; jens.kurth@med.uni-rostock.de; 6 Core Facility Small Animal Imaging, Rostock University Medical Center, D-18057 Rostock, Germany; jan2.stenzel@gmail.com (J.S.); tobias.lindner@med.uni-rostock.de (T.L.)

**Keywords:** hemiparkinsonian rat model, botulinum neurotoxin-A, olfaction, olfactory bulb, behavior, PET/CT, MRI, D_2_/D_3_ dopamine receptor, connectomics, correlation analysis

## Abstract

Olfactory deficits occur as early non-motor symptoms of idiopathic Parkinson’s disease (PD) in humans. The first central relay of the olfactory pathway, the olfactory bulb (OB), depends, among other things, on an intact, functional crosstalk between dopaminergic interneurons and dopamine receptors (D_2_/D_3_R). In rats, hemiparkinsonism (hemi-PD) can be induced by unilateral injection of 6-hydroxydopamine (6-OHDA) into the medial forebrain bundle (MFB), disrupting dopaminergic neurons of the substantia nigra pars compacta (SNpc). In a previous study, we showed that subsequent injection of botulinum neurotoxin-A (BoNT-A) into the striatum can reverse most of the pathological motor symptoms and normalize the D_2_/D_3_R availability. To determine whether this rat model is suitable to explain olfactory deficits that occur in humans with PD, we examined the availability of D_2_/D_3_R by longitudinal [^18^F]fallypride-PET/CT, the density of tyrosine hydroxylase immunoreactivity in the OB, olfactory performance by an orienting odor identification test adapted for rats, and a connectome analysis. PET/CT and immunohistochemical data remained largely unchanged after 6-OHDA lesion in experimental animals, suggesting that outcomes of the 6-OHDA hemi-PD rat model do not completely explain olfactory deficits in humans. However, after subsequent ipsilateral BoNT-A injection into the striatum, a significant 8.5% increase of the D_2_/D_3_R availability in the ipsilateral OB and concomitant improvement of olfactory performance were detectable. Based on tract-tracing meta-analysis, we speculate that this may be due to indirect connections between the striatum and the OB.

## 1. Introduction

Parkinson’s disease (PD) is one of the most prevalent progressive neurodegenerative disorders characterized by the loss of dopaminergic terminals in the striatum and neurons in the substantia nigra pars compacta (SNpc), resulting in cardinal motor symptoms, such as resting tremors, rigidity, bradykinesia, and postural instability [1,2,3,4]. As a prominent non-motor symptom, especially, olfactory deficits (hyposmia, anosmia) occur in more than 90% of PD patients [5,6,7,8,9].

Anosmia or hyposmia may precede the outbreak of motor symptoms by years [5,10,11,12,13]. Despite the high prevalence of olfactory impairment in PD, no causal therapy has yet been proven to be effective in PD-related smell loss [7,11,14,15,16]. PD has been described as a multiple system disorder that is accompanied by disturbances not only in dopaminergic but also in cholinergic, noradrenergic and serotonergic pathway systems [17,18,19,20,21]. Cerebral changes in PD have been described and well documented by the Braak staging which initially involves the vagal dorsal nucleus and the olfactory bulb (OB) [8,22,23,24,25,26]. The clinically dominant motor/stature failure is attributed primarily to the lack of dopaminergic neurons in the SNpc that normally project to the basal ganglia complex of caudate nucleus and putamen (CPu) via the medial forebrain bundle (MFB) [27,28,29,30]. In a well-established animal model, SNpc dopaminergic neurons are destroyed by injection of 6-hydroxydopamine (6-OHDA) into the MFB, causing hemiparkinsonian (hemi-PD) conditions [31,32,33,34].

Since dopamine is a key transmitter in periglomerular inhibitory neurons of the OB [35,36,37,38], we speculate that hemi-PD may also lead to dopamine-associated alterations in the OB causing measurable olfactory impairment.

In PD, dopamine depletion leads to hyperactivity of cholinergic interneurons in the striatum [39,40,41,42]. Botulinum neurotoxin-A (BoNT-A) inhibits the release of acetylcholine in the peripheral nervous system and is also thought to act as a local, non-toxic anticholinergic drug when injected intrastriatally, i.e., into the CPu in hemi-PD rats [43,44,45,46,47,48,49,50,51,52,53,54]. In hemi-PD rats, injection of 1 ng BoNT-A into the DA-depleted CPu significantly diminished apomorphine-induced rotational behavior for at least 3 to 6 months, the effect fading thereafter [43,44,45,46,47,48,49,50,51,52,53,54]. As known from various medical implementations, BoNT-A demonstrates a transient therapeutic effect in hemi-PD rats that lasts up to six months post-injection [43,44,45,46,47,48,49,50,51,52,53,54,55].

Positron emission tomography/computed tomography (PET/CT) using radioligand [^18^F]fallypride enables the detection of the D_2_/D_3_ receptor (D_2_/D_3_R) availability in vivo [56,57,58,59,60]. In a previous study, we applied [^18^F]fallypride PET/CT to analyze the D_2_/D_3_R in the CPu of hemi-PD rats one, three, and six months after BoNT-A or Sham-BoNT-A injection [58]. There was a 23% increase of D_2_/D_3_R availability in the ipsilateral CPu of hemi-PD rats. Subsequently, intrastriatal injection of BoNT-A almost normalized the D_2_/D_3_R availability and reversed apomorphine-induced rotational behavior [58]. These results suggest a therapeutic effect of BoNT-A on the impaired motor behavior of hemi-PD rats by reducing interhemispheric differences of striatal D_2_/D_3_R.

We tested the hypothesis whether a unilateral 6-OHDA lesion of the right MFB is a suitable procedure to induce and study olfactory deficits in rats. We speculated that dopaminergic deafferentation of the CPu could lead to alterations in the expression of D_2_/D_3_R in the OB. Thus, in parallel approaches, D_2_/D_3_R availability was analyzed by dynamic [^18^F]fallypride-PET/CT scans. Each rat was longitudinally scanned for OB D_2_/D_3_R availability 1, 3, and 6 months after intrastriatal BoNT-A or Sham-BoNT-A injection. In a subsequent approach using identical injection procedures, rats were tested for their olfactory abilities in the buried pellet test. 

Additionally, pathway and graph distance analysis in combination with multivariate statistics were used to study the connectivity of the CPu and the OB, i.e., the olfactory-basal ganglia-connectivity (OBG), according to literature results on tract-tracing publications and its resulting possible planar network.

## 2. Results

### 2.1. Drug-Induced Behavior Tests

#### 2.1.1. Apomorphine-Induced Rotation Test

6-OHDA lesion of >95% was examined in apomorphine-induced rotational testing [61,62,63,64]. In the 6-OHDA animal model used here, dopaminergic deafferentation caused a compensatory increase in D_2_/D_3_R availability in the right CPu of hemi-PD rats by 23% [58]. This effect accounts for the motoric effect induced by injection of the D_2_/D_3_R agonist apomorphine [65,66,67,68].

In the present study, after apomorphine injection, non-injected (median −0.00896 min^−1^) and Sham + Sham (median 0.0704 min^−1^) rats did not show obvious rotational behavior. 6-OHDA + Sham rats exhibited strong contralateral rotations with a median of 8.451 min^−1^. In comparison, 6-OHDA + BoNT rats showed significantly decreased rotations (median 1.719 min^−1^; *p* < 0.05).

#### 2.1.2. Amphetamine-Induced Rotation Test

Amphetamine induces the release and inhibits the reuptake of dopamine in the striatum and causes ipsilateral rotation in animals with unilateral nigrostriatal lesions. In rats, amphetamine-induced rotation correlated both with the extent of the TH cell loss and with the degree of striatal dopamine deficiency [69,70,71,72,73].

Following amphetamine injection, non-injected (median 0.370 min^−1^) and Sham + Sham (median 0.436 min^−1^) rats did not show significant rotational behavior. 6-OHDA + Sham rats exhibited strong (median −12.890 min^−1^; *p* < 0.05) clockwise rotations, i.e., in the direction of the lesioned side. Compared with Sham-lesioned hemi-PD rats, 6-OHDA + BoNT rats tended to show fewer rotations (median −9.996 min^−1^; *p* > 0.05).

### 2.2. Hemi-PD Rats Do Not Show Olfactory Deficits, but BoNT-A-Injected Hemi-PD Rats Improve Performance in the Buried Pellet Test

The buried pellet test, originally developed for mice, measures the food motivation aspect of olfaction, testing the ability of hungry (food restricted) animals to detect a palatable piece of sweetened cereal buried under bedding [74]. In this study, the test was adapted for rats for the first time.

**Figure 1:**
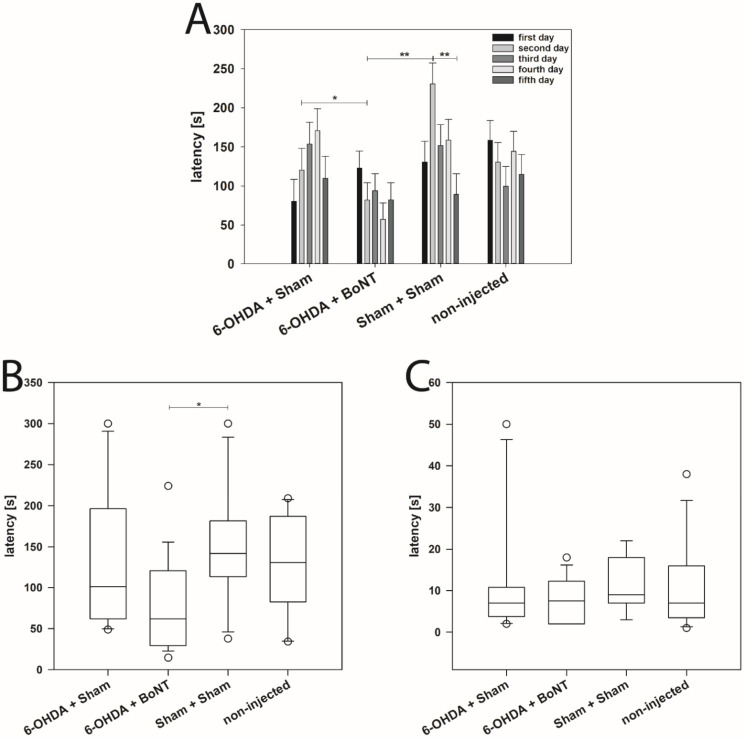
Buried pellet test. (**A**) Latency to find the pellet in the buried pellet test of rats of the four groups during testing days 1 to 5. (**B**) Mean latency of rats of the four groups to find the pellet on days 1 to 5. Rats of the 6-OHDA + BoNT group were significantly faster in the buried pellet test than those of the Sham + Sham group. (**C**) In the surface pellet test, the latency to find the pellet was not significantly different among all four groups. Bar plots are given as means ± SD. Boxplots depict groups graphically by displaying the descriptive statistical parameters: median, upper, and lower quartiles, and outliers (circles) that lie outside the 10th and 90th percentiles (whiskers). Asterisks indicate significant differences after performing all multiple comparison procedures in pairs (Holm-Sidak or Dunn’s method; * *p* < 0.05, ** *p* < 0.01).

Rats were tested daily on 5 consecutive days. As seen in Figure 1A, rats of neither group showed a learning effect throughout the experiments; the time (latencies) to find and start eating the pellet varied considerably between the days and the rats of the various groups. Means of latencies were calculated for each group (Figure 1B). The latencies of non-injected (129.55 ± 17.56 s) and Sham + Sham (152.13 ± 20.95 s) rats did not differ significantly (*p* = 0.658). The respective values of 6-OHDA + Sham rats (127.02 ± 25.61 s) correspond roughly to those of the non-injected group. Thus, hemi-PD rats did not show significant olfactory deficits. Compared with Sham-treated hemi-PD rats (127.02 ± 25.61 s), 6-OHDA + BoNT rats (78.97 ± 13.47 s) tended to find the buried pellet faster (*p* = 0.253). However, the difference in latency between the 6-OHDA + BoNT rats and the Sham + Sham group was significant (*p* = 0.033) (Figure 1B).

On testing day 6, the latencies were measured accordingly, but the pellet was now placed on the surface of the bedding (surface pellet test [74]) (Figure 1C). In the surface pellet test, which was performed to exclude possible motor disorders or alterations in feeding motivation, the latencies to find the pellet did not differ between any of the four experimental groups (Figure 1C). Median latencies were 7.00 s in the non-injected rats, 9.00 s in the Sham + Sham rats, 7.00 s in the 6-OHDA + Sham rats, and 7.50 s in the 6-OHDA + BoNT rats. Neither experimental group is likely to have impaired visual perception or spontaneous motor behavior in the given test situation.

### 2.3. There Is No Densitometric Interhemispheric Difference of TH-Immunoreactivity in the OB

**Figure 2:**
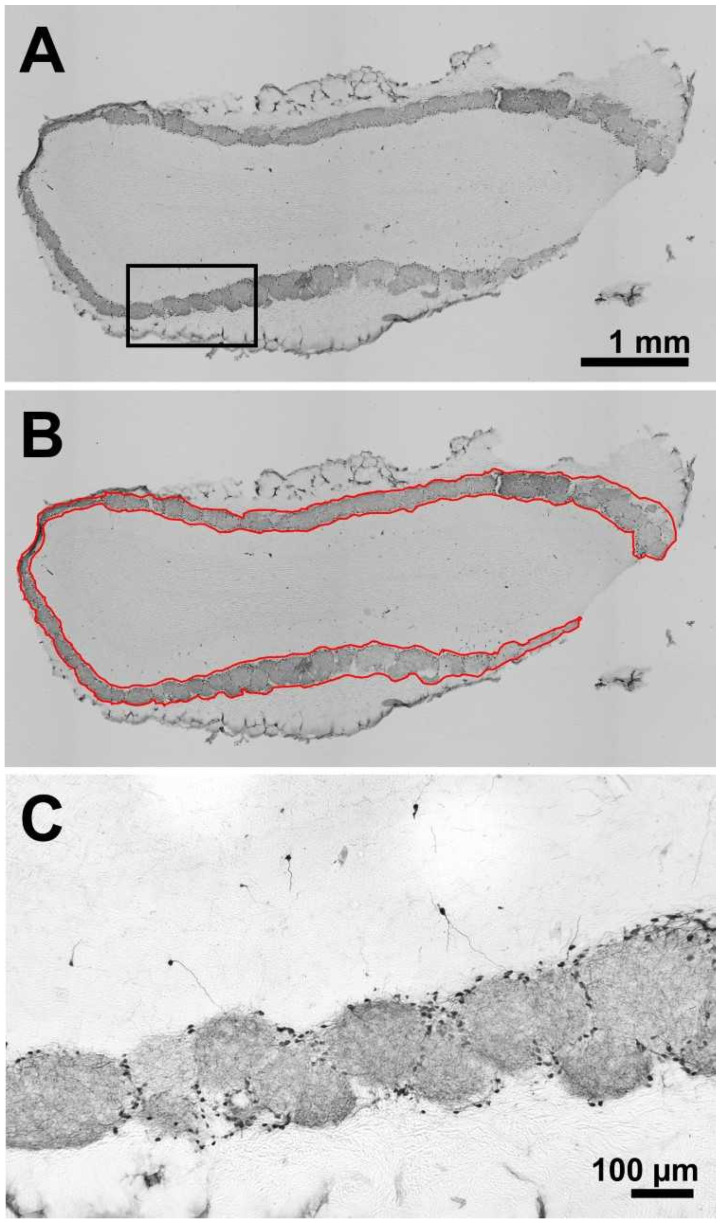
Frontal section of an OB used for densitometry (**A**–**C**). The section shows a left OB of a hemi-PD rat unilaterally lesioned into the right MFB, immunohistochemically reactive to TH (**A**,**C**). In (**B**), the glomerular layer is outlined (red) to define the region of interest for the evaluation algorithm. (**C**) Higher magnification marked by a rectangle in (**A**).

The glomerular layer with its tyrosine hydroxylase (TH)-positive cells and nerve fibres, which is wrapped around the outer area of the OB similar to a band, can be seen clearly (Figure 2A–C). The stained specimens were scanned in 8-bit grayscale mode, and the optical densities of the glomerular layer of both the left and right OB were determined in the 4 animal groups. 

**Figure 3:**
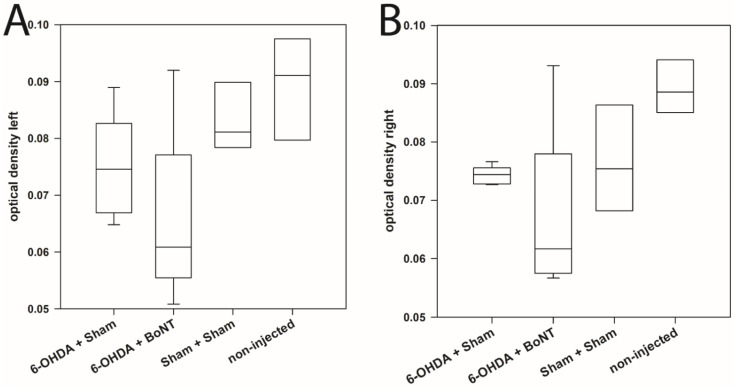
Densitometric measurement of TH-immunoreactivity of the glomerular layer of the left (**A**) and right (**B**) OB in four experimental groups. Boxplots depict groups graphically by displaying the descriptive statistical parameters: median, upper, and lower quartiles.

Densitometric analysis of the glomerular layer immunohistochemically reactive to TH of OB in both hemispheres revealed no significant interhemispheric differences in any of the 4 animal groups (Figure 3A,B): The median optical densities of the glomerular layer of the left and right OB of the non-injected rats were 0.091 and 0.089, of the Sham + Sham rats were 0.081 and 0.075, of the 6-OHDA + Sham rats were 0.075 and 0.074, and of the 6-OHDA + BoNT rats were 0.061 and 0.062. In addition, no significant differences between the optical densities of the glomerular layers were found between the respective values of the different experimental groups (*p* > 0.05) (Figure 3A,B).

### 2.4. Olfactory Bulb D_2_/D_3_R Availability—Receptor Upregulation after Ipsilateral Intrastriatal BoNT-A Injection 

Olfactory bulb D_2_/D_3_R availability was analyzed in controls (Sham + Sham, n = 9), Sham-BoNT-A-injected hemi-PD rats (6-OHDA + Sham, n = 9) and BoNT-A-injected hemi-PD rats (6-OHDA + BoNT-A, n = 10) by dynamic [^18^F]fallypride-PET/CT scans. Each rat was longitudinally scanned for OB D_2_/D_3_R availability 1, 3, and 6 months after BoNT-A or -Sham-BoNT-A injections.

**Figure 4:**
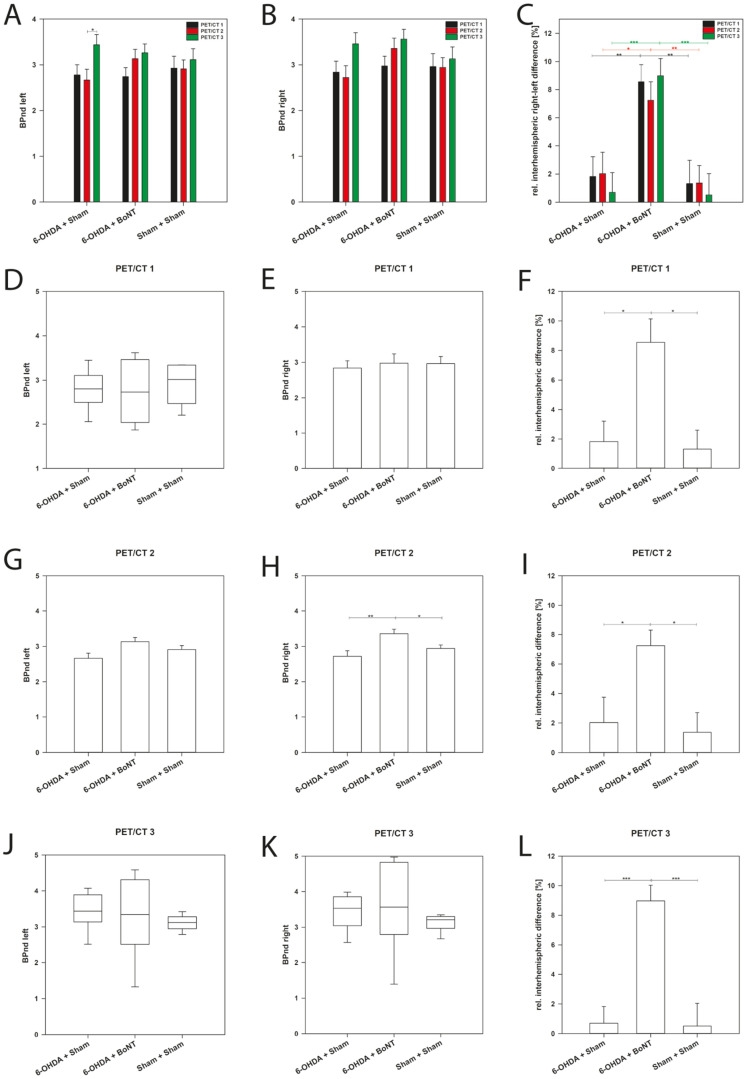
Absolute Binding potential (BP_nd_) of D_2_/D_3_R in the left and right OB for each experimental group at time point of PET/CT1, PET/CT2, and PET/CT3, and relative interhemispheric (right/left) difference (%). (**A**–**C**) means of the PET/CT1—3 measurements of the BP_nd_ of the left and right OB and the calculated relative interhemispheric right/left difference (%), (**D**–**F**) respective values of the PET/CT 1 (1 month post-BoNT-A or Sham), (**G**–**I**) respective values of the PET/CT 2 (3 months post BoNT-A or Sham), and (**J**–**L**) values of the PET/CT 3 (6 months post-BoNT-A or Sham) of 6-OHDA + Sham, 6-OHDA + BoNT, and Sham + Sham groups. Bar plots are given as means ± SD. Boxplots graphically depict groups by displaying the descriptive statistical parameters: median, upper, and lower quartiles, and outliers (circles) that lie outside the 10th and 90th percentiles (whiskers). Asterisks indicate significant differences after performing all multiple comparison procedures in pairs (Holm-Sidak or Dunn’s method; * *p* < 0.05, ** *p* < 0.01, *** *p* < 0.001).

For quantification, the simplified reference tissue model 2 (SRTM2) was applied, and the non-displaceable Binding Potential (BP_nd_), which is used to measure the receptor transporter density [59], was estimated separately for the left and right OB in each experimental group at each time point. Remarkably, there were no significant differences between the absolute values of the BP_nd_ of the Sham + Sham and the 6-OHDA + Sham rats (Table 1; Figure 4A,B,D,E,G,H,J,K). However, the right OB of BoNT-treated hemi-PD rats in PET/CT 2 exhibited significant results, about 8.5% higher BP_nd_ values compared with both Sham + Sham and 6-OHDA + Sham rats (Table 1, Figure 4H). This may speak to BoNT-A-induced D_2_/D_3_R upregulation in the OB of hemi-PD rats after ipsilateral intrastriatal BoNT-A injection.

Calculation of relative interhemispheric differences of BP_nd_ values revealed more differentiated results. In controls (Sham + Sham), no significant relative interhemispheric right/left differences were found: In PET/CT 1, mean BP_nd_ values of the left OB (2.93 ± 0.26) and the right OB (2.96 ± 0.29) showed a small, non-significant relative interhemispheric difference of 1.32 ± 1.66% (Table 1, Figure 4C,F). Respectively, relative interhemispheric difference in the same range were found in PET/CT 2 and PET/CT 3 (Table 1, Figure 4C,I,L).

PET/CT1 of 6-OHDA + Sham rats also had a small relative interhemispheric right/left difference in BP_nd_ values (1.83 ± 1.40%) (Table 1, Figure 4C,F), as did PET/CT 2 (2.03 ± 1.51%) (Table 1, Figure 4C,F) and PET/CT 3 (0.70 ± 1.40 %) (Table 1, Figure 4C,I,L).

However, contrasting results were found in the 6-OHDA + BoNT group, showing a significant relative interhemispheric right/left difference in BP_nd_ values at each measurement time point: PET/CT 1 (8.54 ± 1.24%), PET/CT 2 (7.24 ± 1.31%), and PET/CT 3 (8.98 ± 1.24%) (Table 1, Figure 4C,F,I,L), compared with both the Sham + Sham and the 6-OHDA + Sham rats (PET/CT 1: *p* = 0.010 and *p* = 0.012; PET/CT 2: *p* = 0.013 and *p* = 0.036; PET/CT 3: both *p* < 0.001). Obviously, upregulation of D_2_/D_3_R occurred in the OB of the dopamine-depleted hemisphere, which was additionally injected with BoNT-A into the ipsilateral striatum.

### 2.5. Connectomics—There Are No Direct Pathways between the Main Olfactory Bulb and Basal Ganglia

**Figure 5:**
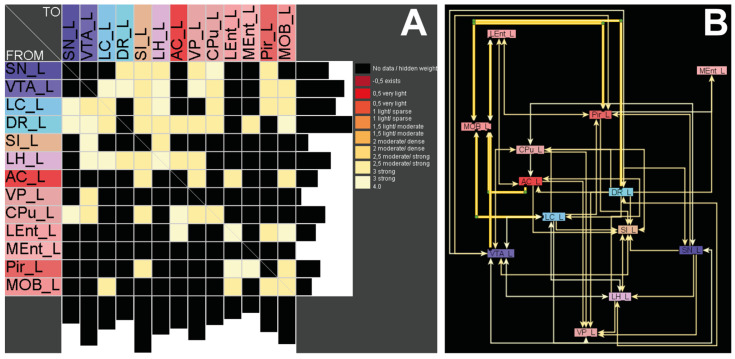
(**A**) Bilateral connectivity of the CPu and the MOB based on a tract-tracing meta-study using neuroVIISAS (neuroviisas.med.uni-rostock.de). (**B**) Planar network visualization of the connection matrix shows several indirect CPu-MOB connections, but no direct connections. Abbreviations: from: SN_L, left substantia nigra; VTA_L, left ventral tegmental area; LC_L, left locus coeruleus; DR_L, left dorsal raphe nucleus; SI_L, left substantia innominate; LH_L, left lateral hypothalamus; AC_L, left amygdaloid complex; VP_L, left ventral pallidum; CPu_L, left caudate putamen (striatum), LEnt_L, left lateral entorhinal cortex, MEnt_L, left medial entorhinal cortex, Pir_L, left piriform cortex; MOB_L, left main olfactory bulb.

Based on the neuroanatomical components of the basal ganglia complex, as well as the main olfactory bulb (MOB), we first tested whether a direct neuronal connection exists between the MOB and CPu. This was found neither ipsilaterally nor contralaterally. Using the pathway analysis function of neuroVIISAS, all shortest pathways across intermediate regions between the CPu and the MOB were determined, and then filtered with respect to their geometric distance, as well as connection weights: The regions involved in Figure 5A,B represent the strongest connectivity between the MOB and CPu. The results were represented first as an adjacency matrix (Figure 5A). The tenses of the adjacency matrix contain the same regions and the same region sequences as the columns, so it is a square matrix. The rows indicate the source regions of the neural connections, and the columns indicate the regions that receive the connections (target regions) (Figure 5A). The connection weights represent semi-quantitative values in analogy to the data in the original research literature. Second, data are represented as a planar weighted and rectified orthogonal graph (Figure 5B). There is no direct connection from the CPu to the MOB. All connections from CPu to MOB have at least one intermittent region. With regard to the frequency of observations in tract-tracing publications, there are three possibly modified pathways: CPu→dorsal raphe nucleus→MOB, CPu→ventral tegmental area→MOB, CPu →amygdala complex→MOB are the most promising candidates for the BoNT-A-induced D_2_/D_3_R increase in hemi-PD rats (Figure 5B).

### 2.6. Correlating Buried Pellet Test, Apomorphine- and Amphetamine-Induced Rotations, and Optical Densites of Glomerular Layers of the Left and Right OB

**Figure 6:**
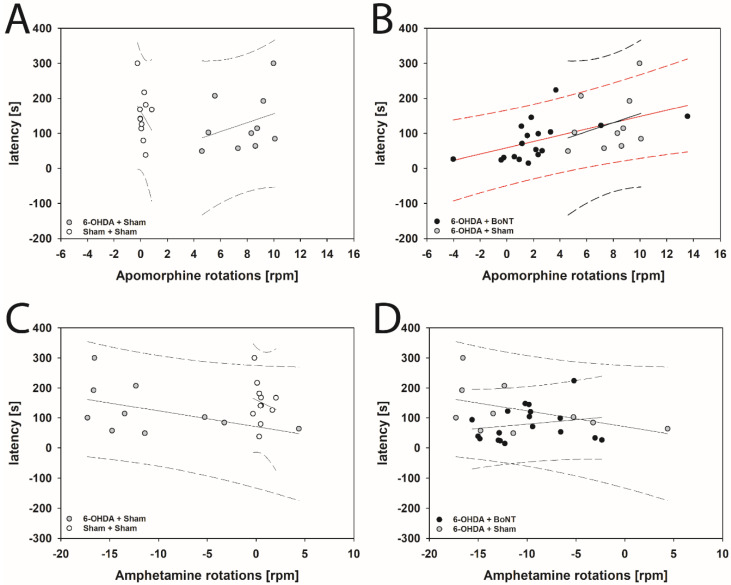
The latencies to find the pellet in the buried pellet test correlate with the apomorphine-induced rotations of rats of the 6-OHDA + Sham and Sham + Sham groups (**A**), and the 6-OHDA + BoNT and 6-OHDA + Sham groups (**B**). (**C**) The latency data correlate with the respective amphetamine-induced rotations of rats in the 6-OHDA + Sham and Sham + Sham groups and (**D**) of the 6-OHDA + BoNT and 6-OHDA + Sham groups. Only in rats of the 6-OHDA + BoNT group did (**B**) the latency to find the pellet significantly correlate with decreasing clockwise apomorphine-induced rotations. Regression lines are displayed as solid lines and prediction intervals as dashed lines. Red-colored regression line (**B**) and prediction interval lines indicate significant regression (*p* < 0.05).

Prior to the buried pellet test, all animals were subjected to apomorphine- and amphetamine-induced rotation tests approximately two weeks after injection of the vehicle or BoNT-A (Figure 6A–D). With the correlation analysis, we checked whether there was a dependence of parameters measured in the buried pellet test on apomorphine- and amphetamine-induced rotations. If so, the results of the drug-induced rotations could possibly predict measures to indicate olfactory deficits in hemi-PD rats. In addition, we analyzed the effect of a unilateral intrastriatal BoNT-A injection in hemi-PD rats (Figure 6B,D).

The buried pellet test showed that the latency to find the pellet was significantly correlated with apomorphine-induced rotations in rats in the 6-OHDA + BoNT group: rats with shorter latencies had fewer clockwise rotations (rs = 0.709; *p* = 0.001; Figure 6B). The correlation is even significant if the two outliers with the lowest and highest rotation values are excluded from the analysis (rs = 0.629; *p* = 0.0087). 

In correlation analysis, rats of the 6-OHDA + Sham and Sham + Sham groups lacked significant dependence of latency and apomorphine-induced rotation (*p* = 0.371 and *p* = 0.416; Figure 6A,B). No significant correlations were found between the latencies of rats in the 6-OHDA + BoNT, 6-OHDA + Sham, and Sham + Sham groups on amphetamine-induced rotations (*p* = 0.293, *p* = 0.227, and *p* = 0.714; Figure 6C,D).

The latency to find the pellet of rats in the 6-OHDA + BoNT, 6-OHDA + Sham, and Sham + Sham groups after 6-OHDA lesion, and also after BoNT-A or vehicle injection, did not correlate with the optical densities of the glomerular layers of the left and right OB (Supplementary Materials, Figure S1A–D).

No significant correlations were found between the optical densities of the glomerular layers of the left OB with appropriate apomorphine- and amphetamine-induced rotations in 6-OHDA + Sham, 6-OHDA + BoNT, and Sham + Sham groups (Supplementary Materials, Figure S2A–D).

Correspondingly, also the optical densities of glomerular layers of the right OB in the 6-OHDA + Sham, 6-OHDA + BoNT, and Sham + Sham groups did not significantly correlate with respective apomorphine- and amphetamine-induced rotations (Supplementary Materials, Figure S3A–D).

## 3. Discussion

Here, we present a longitudinal study of changes in olfactory bulb D_2_/D_3_R availability in the 6-OHDA-induced hemi-PD rat model and results of an olfactory detection test.

Hemi-PD rats did not show significant olfactory deficits in the buried pellet test. However, 6-OHDA + BoNT rats found the hidden pellet significantly faster than animals of the control group. Densitometric analysis of TH-immunoreactivity in the glomerular layer of the OBs of both hemispheres showed no significant interhemispheric differences in any of the 4 animal groups. When right and left D_2_/D_3_R availability in the OBs were measured, the right OB of BoNT-A-treated hemi-PD rats 6 weeks, 3 months, and 6 months (PET/CT 1-3) after BoNT-A injection showed significant, about 8.5% higher, BP_nd_ values in the right OB compared with both Sham + Sham and 6-OHDA + Sham rats. Apparently, BoNT-A injected intrastriatally induced D_2_/D_3_R upregulation in the OB of hemi-PD rats.

Consistent with the hypothesis that the CPu might affect the OB, resulting in olfactory deficits, a tract-tracing meta-study revealed no direct neuronal connection, based on more than one publication, between the OB and CPu in rats. However, promising candidates for a D_2_/D_3_R regulation between the CPu and the OB are intermittent pathways via the dorsal raphe nucleus or the ventral tegmental area or the amygdala complex. Testing for correlations between the parameters examined in this study revealed that the latency to find the buried pellet was strongly correlated with apomorphine-induced rotations of rats in the 6-OHDA + BoNT group: rats with shorter latencies had fewer anti-clockwise rotations. This indicates that striatal injection of BoNT-A improves olfactory performance.

### 3.1. Olfactory Deficits in PD

Among all non-motor features in PD, olfactory deficits are the most prominent changes [21,24,75,76,77]. The OB is the first important relay in the central olfactory system that receives neural input not only from the olfactory mucosa but also from multiple brain regions [78,79,80,81,82]. The OB consists of a population of about 10% dopaminergic interneurons, primarily located within the glomolerular layer [35,76,83,84,85,86,87]. Dopaminergic neurons build synapses with mitral cells, which, in turn, express serotonin, noradrenaline and dopamine receptors [88], as well as modulate odor information processing [36,89,90,91] and behavioral odor discrimination [92,93]. Olfactory deficits in PD have been intensively studied almost exclusively in transgenic mouse models [94,95,96,97,98,99,100,101,102,103] or in the MPTP mouse model [104,105,106]. This study is the first one to analyze olfactory abilities in a 6-OHDA rat model.

### 3.2. Hemi-PD Rats Show No Olfactory Deficits in the Buried Pellet Test

Although a few studies have described the 6-OHDA animal model with unilateral or bilateral lesion of the SN or the OB, or both [107,108,109,110,111,112,113], no olfactory behavior tests have been performed. A few studies using these tests have been done, however, only in differently designed 6-OHDA or MPTP mouse models [76,101,104,114,115] or in mice with neurodegenerative diseases, such as Niemann-Pick Disease Type C1 [116]. Outcomes of our buried pellet test demonstrated that rats of neither group showed olfactory deficits. Remarkably, intrastriatal BoNT-A injection in hemi-PD rats significantly improved olfactory performance in the buried pellet test in 6-OHDA + BoNT rats compared with the Sham + Sham group. Compared with the Sham-lesioned hemi-PD rats, 6-OHDA + BoNT rats tended to find the buried pellet faster (but not significantly). This correlated well with our results obtained from PET/CT experiments on D_2_/D_3_R availability in the OBs of the same animal group and highlights the importance of the dopaminergic balance in the OB.

Taken together, hemi-PD rats showed no olfactory deficits. However, intrastriatal BoNT-A injection in hemi-PD rats significantly improved latency in the buried pellet test in 6-OHDA + BoNT rats compared with the Sham + Sham group and, tendentially, also compared with the 6-OHDA + Sham rats.

### 3.3. Dopamine as a Key Transmitter in Processing of Olfactory Information

In order to understand functional changes in the OB after unilateral lesion of the MFB, we studied the behavior of both dopaminergic interneurons by immunohistochemistry and the correspondent availability of D_2_/D_3_R using PET/CT.

Dopamine is a key transmitter for processing olfactory information in the glomerular layer of the OB, which is a crosstalk between dopaminergic interneurons and D_2_ receptors in the terminals of axons of olfactory and in presynaptic elements of the glomerular neuropil [117]. Thus, dopamine could act in glomerular circuits through presynaptic mechanisms mediated by D_2_ receptors. Correspondingly, a high density of D_2_ receptors was anatomically localized especially in the glomerular layer [118,119,120,121,122,123,124,125]. All structures related to olfaction show high numbers of D_2_ and D_1_ receptors [126].

#### 3.3.1. Hemi-PD Rats Do Not Show Altered TH-Immunoreactivity in the Glomerular Layer of the OB

Synuclein-immunopositive Lewy bodies and Lewy neurites in the OB have been reported in very early stages of PD [24]. An increased number of olfactory dopaminergic glomerular cells in PD was first reported by Huisman and colleagues [127]. Since glomerular dopaminergic TH-immunoreactive interneurons in OB release dopamine and GABA, which would inhibit glutamatergic neurotransmission from receptor neurons to mitral cells [37,128,129], it has been suggested that the increased number of inhibitory TH-immunoreactive interneurons might cause hyposmia in PD [127,130]. In addition, the increase of dopamine levels in the OB possibly explains why, in PD, olfaction does not improve with levodopa therapy [131]. The increase in the number of dopaminergic neurons in the OB could reflect a compensatory mechanism, created by the early degeneration of other neurotransmitter systems, and might contribute to the olfactory dysfunction in PD patients. The hypothesis arose that this modification could be a general characteristic for other neurodegenerative disorders involving cholinergic, noradrenergic and serotonergic denervation. Cholinergic [132,133] and noradrenergic [134,135] centrifugal effects on the OB exert an inhibitory effect on the mitral cell layer of OB, while serotonergic input can activate periglomerular neurons to release GABA [136]. Therefore, a dysfunction of the centrifugal sense of smell input from an early degeneration of these systems could cause an inhibitory imbalance that could be corrected by increasing the number of TH-immunoreactive periglomerular neurons. In order to evaluate whether lesioning of the SNpc via MFB injection of 6-OHDA is followed by changes in dopaminergic parameters in the rat OB, densitometric analysis of the glomerular layer of the OB, stained immunohistochemically against TH, was carried out in addition to the availability of the D_2_/D_3_R. Densitometric analysis in both hemispheres in our study revealed no significant interhemispheric differences in any of the 4 animal groups. In addition, no significant differences were found between the optical densities of the glomerular layers between the respective values of the different experimental groups. Thus, our study adds a further result to the conflicting reports on the TH-immunoreactivity in the OB of PD patients or rodent PD models. There are only two studies whose results demonstrated increased numbers of TH-immunoreactive neurons after unilateral lesion trials in rats. Winner et al. [96] described changes 6 weeks after unilateral lesion of the MFB, and Voronkov et al. [110] reported an increase after unilateral SN lesion. The latter authors interpreted this as a possible compensatory process, and argued that a more pronounced dopamine-related inhibitory effect in the glomeruli of the OB could contribute to the development of hyposmia in PD. Olfactory behavior tests were not performed. An increased number of olfactory dopaminergic glomerular cells in the autoptic material of PD patients was first reported by Huisman et al. [127,130] and later observed in Alzheimer’s disease and frontotemporal dementia patients when compared with age-matched controls [137]. Our results are supported by Ilkiw et al. [112], who found no increase in TH-immunoreactive periglomerular cells compared with controls, and, interestingly, also no changed measurements in the olfactory discrimination task after 6-OHDA lesion of the SN. Controversial results on the effect of unilateral lesion have also been reported in mice. Zhang et al. [76] observed no difference in the number of dopaminergic interneurons in the OB after injection in the SNpc, whereas Sui et al. [138], and Chiu et al. [139] reported a significant increase.

#### 3.3.2. Intrastriatal BoNT-A Injection Led to an Upregulation of D_2_/D_3_ Receptor Availability in the Ipsilateral OB of Hemi-PD Rats

We further analyzed the availability of the D_2_/D_3_R of the OB after unilateral 6-OHDA lesion into the right MFB, followed by unilateral intrastriatal BoNT-A or vehicle injection in male Wistar rats using [^18^F]fallypride-PET/CT scans. We focused on olfaction-related parameters in hemi-PD rats with unilateral lesion of the SNpc and subsequent intrastriatal BoNT-A injection. Based on previously reported results on [^18^F]fallypride-PET/CT in the CPu [58], we speculated that dopaminergic deafferentation induced by unilateral injection of 6-OHDA into the right MFB of the CPu via destruction of the SN may also lead to alterations in the expression of dopamine D_2_/D_3_R in the OB. Moreover, altered proliferation of adult neuronal progenitor cells in the subventricular zone and their migration to and positioning and differentiation in various neuron types in the OB seen in hemi-PD mouse models could result in changes in D_2_/D_3_R [138,139]. Our study also revealed that the induction of hemi-PD following 6-OHDA injection into the MFB did not alter the D_2_/D_3_R availability of the OBs. Surprisingly, although studied in other brain regions, data on the quantification of D_2_/D_3_R in the OB are not evaluable in PD patients [140,141] nor in animal models of PD [142,143].

The intrastriatal BoNT-A injection in hemi-PD rats, however, resulted in an 8.5% increase of the D_2_/D_3_R availability of the ipsilateral OB and is accompanied by a better olfactory test performance. The reasons for this, however, remain unclear.

The only significant correlation in the various parameters measured in the present study were found in the 6-OHDA + BoNT rats between the latency to find the buried pellets and apomorphine-induced rotations: the faster the pellets were found by the rats, the fewer rotations they made. The beneficial effect of intrastriatal BoNT-A in hemi-PD rats with respect to the reduction of apomorphine-induced rotational behavior could be explained by modifications in basal ganglia circuitry and changes in densities of dopaminergic and cholinergic receptors in the CPu [43,45,46,47,52,53,58,144,145]. Rats of the 6-OHDA + Sham and Sham + Sham groups lacked any significant dependence of latency and apomorphine- or amphetamine-induced rotations. Therefore, in hemi-PD rats, analysis of motor deficits is apparently not predicative for changes of olfaction performance.

The significantly increased interhemispheric difference of approximately 8% in D_2_/D_3_ receptor availability in the ipsilateral OB of BoNT-A-treated hemi-PD rats is of biological relevance. Even, at first glance, small but significant differences in receptor density have been shown to be functionally significant. For example, in a study on circling rats, a significant difference in adenosine A1 receptor binding site density was found between ci2 rat mutants and wild types in the range of 10% in motor areas [146]. In wild type rats (LEW/Ztm strain), the CPu in the left hemisphere had a significantly (5%) lower density of kainate receptor binding sites than in the right hemisphere [147]. In aged Fischer 344/Brown Norway rats that differed in retention performance in a water maze reference memory task, superior and inferior learners had significantly different binding site densities of various receptors in the range of 10–15% [148]. In different mouse models with PD-associated gene mutations, quantitative multi-receptor studies revealed significant differences in receptor-binding sites in various brain areas, ranging from 9% to 42% [142,143].

### 3.4. Connectomics Reveal Indirect Projections between the OB and the CPu 

The results of the PET/CT study may account for a compensatory D_2_/D_3_R increase in hemi-PD rats which were injected with BoNT-A into the dopamine-deprived CPu and could be explained most probably by a functional connectivity of the CPu and the OB. To clarify possible connectome pathways, olfactory connectome analysis provided evidence that both direct and indirect axonal connections may act as the structural backbone for regulating regional receptor expressions. We found that the glomerular layer of the OB had unilateral and reciprocal interconnectivity with the VTA, and the projection from OB to CPu is non-reciprocal. VTA is reciprocally connected with SN and CPu. This indirect connectivity of the OB may be the reason for D_2_/D_3_R upregulation after MFB lesion with subsequent intrastriatal BoNT-A injection. According to the hypothesis that the CPu might affect the OB and, thereby, possibly cause olfactory deficits, we found no direct neuronal connection between the OB and CPu in the literature. However, promising candidates for a D_2_/D_3_R regulation between the CPu and the OB are intermittent pathways via the dorsal raphe nucleus, the ventral tegmental area or the amygdala complex. In addition, BoNT-A may be transported in anterograde and retrograde directions and also transsynaptically [149,150], as demonstrated in the visual system of mice [151,152] and in the spinal cord of rats, as central effects of intramuscularly administered BoNT [153].

However, the molecular basis of the positive effect of intrastriatal BoNT-A application on D_2_/D_3_R density in the ipsilateral olfactory bulb has not been fully elucidated. Given the indirect morphologic connection between CPu and OB, the interhemispheric difference in D_2_/D_3_R density in the OB appears to be unexpectedly high compared with a difference of approximately 25% within the CPu [55]. We hypothesize that an interhemispheric difference in D_2_/D_3_R availability would have been even more pronounced if the OB had been injected directly.

## 4. Materials and Methods

### 4.1. Animals

**Figure 7:**
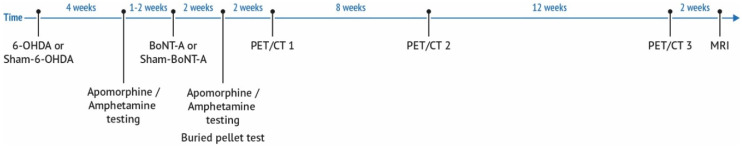
Timeline of the study design. Hemi-PD was induced by 6-OHDA injection into the right MFB. Controls received a Sham-6-OHDA injection. The degree of dopaminergic cell loss was verified with apomorphine- and amphetamine-induced behavioral testing. Six weeks after the 6-OHDA or Sham-6-OHDA injection, rats obtained BoNT-A or Sham-BoNT-A injection into the ipsilateral CPu. The positive effect on the motor behavior of BoNT-A was then controlled in rotation tests. To verify alterations of the olfactory ability, as well as the effect of the intrastriatal BoNT-A injections on the olfactory function in hemi-PD rats, the buried pellet test was conducted. Subsequently, each rat was scanned by [^18^F]fallypride-PET/CT analysis one, three, and six months post-BoNT-A or post-Sham-BoNT-A injection. A final MRI scan was performed as an anatomical reference for PET/CT imaging.

Fifty-one young adult, 3 month-old, male Wistar rats (strain Crl:WI BR, Charles River Wiga, Sulzfeld, Germany) weighing 295–310 g at the time of the first surgery were used. Animals were housed in standard cages in a temperature-controlled room (22 °C ± 2 °C) under a fixed 12 h light/dark cycle and had free access to food and water ad libitum. All procedures used complied with the guidelines on animal care. All experiments (Figure 7) were approved by the State Animal Research Committee of Mecklenburg-Western Pomerania (LALLF M-V 7221.3-1.1-003/13 from 26 April 2013; LALLF M-V/7221.3-1-005/16 from 3 August 2016; LALLF M-V 7221.3-1-056/18, 26 November 2018).

### 4.2. Induction of Hemiparkinsonism

All surgeries were carried out under aseptic conditions and deep anesthesia by intraperitoneal injection of a mixture of ketamine (50 mg/kg^−1^ BW) and xylazine (4 mg/kg^−1^ BW). To induce a unilateral degeneration of dopaminergic neurons in the SNpc and hemiparkinsonian symptoms, a unilateral injection of 24 µg 6-OHDA (Sigma-Aldrich, St. Louis, MO, USA) dissolved in 4 µL of 0.1 M citrate buffer was performed over 4 min via a 26 gauge 5 µL Hamilton syringe into the right MFB using a David Kopf stereotactic frame. The Sham hemi-PD rats received 4 μL of the 0.1 M citrate buffer. Thereafter, the needle was left in place for another 5 min to avoid reflux. The injection coordinates with reference to bregma were: AP = −2.3, L = 1.5 to the right, V = −9.0 [154].

### 4.3. Injection of BoNT-A into the Striatum

Hemi-PD rats underwent a second stereotactic surgery six weeks after the 6-OHDA lesion and were treated with either BoNT-A (n = 18) or vehicle (n = 10). 2 × 1 µL BoNT-A solution (Lot No. 13028A1A; List, Campbell, CA, USA, purchased via Quadratech, Surrey, UK; BoNT-A dissolved in phosphate-buffered saline (PBS) supplemented with 0.1% bovine serum albumin (BSA)) was injected with a total dose of 1 ng BoNT-A into the right CPu at two sites [43,44,45,46,48,49,50,52,53,54,144]. Sham-BoNT-A rats received 2 × 1 µL PBS + 0.1% BSA. The respective coordinates of BoNT-A or Sham-BoNT-A injections with reference to bregma were: AP = +1.3/−0.4 mm, L = 2.6/3.6 mm to the right, and V = −5.5 mm [154]. At each injection site, 1 µL (= 0.5 ng) of BoNT-A or Sham-BoNT-A solution was delivered over 4 min using a 26-gauge 5 µL Hamilton syringe.

### 4.4. Behavioral Testing

#### 4.4.1. Drug-Induced Rotation Tests (Apomorphine, Amphetamine)

##### Apomorphine-Induced Rotation Test

The success of the 6-OHDA lesion was verified by the apomorphine-induced rotation test according to Ungerstedt and Arbuthnott [155] four weeks following 6-OHDA or vehicle injection into the right MFB. Apomorphine (Teclapharm, Germany) was injected i. p. (0.25 mg/kg), and, 5 min after the injection, the registration of the animals’ turns over 40 min started in a self-constructed automated rotometer device modified in accordance with Ungerstedt and Arbuthnott [155] and Schwarting and Huston [156]. Rotations were analyzed as complete 360° turns, and mean rotations per minute (rpm) were calculated (anti-clockwise: +, clockwise: −). A further apomorphine-induced rotation test was carried out 4 weeks after the ipsilateral intrastriatal injection of BoNT-A or vehicle. 

##### Amphetamine-Induced Rotational Test

Three days after the apomorphine testing, rats underwent the amphetamine-induced rotational behavior test [43,44,45,61]. Animals were intraperitoneally injected with D-amphetamine sulphate (2.5 mg/kg, dissolved in 0.9% NaCl, Sigma Aldrich, München, Germany) and, after a waiting time of 15 min, were monitored for 60 min as described above.

#### 4.4.2. Buried Pellet Test

Non-injected (naïve controls, n = 12), controls (Sham 6-OHDA + Sham BoNT-A, further on named Sham + Sham, n = 11), Sham BoNT-A-injected hemi-PD rats (6-OHDA + Sham BoNT-A, further named 6-OHDA + Sham, n = 10), and BoNT-A-injected hemi-PD rats (further named 6-OHDA + BoNT, n = 18) were used for this study.

To verify alterations of the olfactory ability, as well as the effect of the intrastriatal BoNT-A injections on the olfactory function in hemi-PD rats, the buried pellet test according to Lehmkuhl et al. [74] as adapted to rats was conducted.

Before testing, animals were food-restricted for 3 days (food was available for 1 h per day) and thereafter maintained at about 90% of free-feeding body weight during testing procedures [157]. One week prior and during food restriction, each tested rat was accustomed to a piece of sweetened cereal pellet later to be buried (Honey Bsss Loops, Kellogg, Munich, Germany) and, therefore, received 2 pieces of the pellets every day.

On all testing days, rats were habituated to the testing room and, 1 h before the test, were kept in their home cage without a water bottle. For the first 5 testing days (buried pellet test), freshly cleaned testing cages (Makrolonbox typ IV, UNO BV, Zevenaar, Netherlands) were prepared with ~3 cm clean bedding, and one pellet was buried 0.5 cm below in one corner of the cage. Importantly, every day the pellet was buried in a different spot in the cage for each trial, and the testing cage and experimenter’s gloves were changed after each animal. For testing, each rat was removed from its home cage and placed in the center of the test cage; the latency time was measured until the rat uncovered the pellet and began eating it. If a rat did not find the pellet within the predetermined time of 300 s, the experiment was terminated, and a latency of 5 min was recorded. Additionally, the experimenter removed the pellet from the bedding, and the rat was allowed to eat it.

On testing day 6, the test was repeated using the same scheme, but now the pellet was placed on the surface (surface pellet test); the time when the rat found and started eating the pellet was recorded.

All trials were videotaped, and the latencies on testing days 1–5 (buried pellet test) and on testing day 6 (surface pellet test) were measured and expressed as means ± SEM.

### 4.5. TH-Immunohistochemistry of the Olfactory Bulb

Rats were killed with an overdose of ketamine/xylazine, and then the body circuit was perfused transcardially with 50 mL of 4 °C cold 0.9% saline, followed by 400 mL of 4% paraformaldehyde (in phosphate-buffered saline (PBS), 0.1 M, pH 7.4). The brains were immediately removed from the skull and postfixed overnight at 4 °C in 4% paraformaldehyde solution. Subsequently, brains were cryoprotected at 4 °C for 48 h in 20% sucrose solution and snap frozen in −50 °C cold isopentane. The brains were stored at −80 °C. Frontal 30 μm-thick brain slices were serially cut with a cryostat (Leica, Germany). Histological sections of the OB were immunohistochemically reacted with an antibody against tyrosine hydroxylase (TH). For this purpose, the sections were washed in PBS, endogenous peroxidases were blocked using 3% hydrogen peroxide solution, and non-specific binding sites were blocked using horse serum. Primary antibody incubation (monoclonal, anti-TH, mouse, 1:1000, Sigma-Aldrich, St. Louis, MO, USA) was performed overnight at 4 °C. Secondary antibody incubation (polyclonal, anti-mouse, horse, 1:200, Vector Laboratories, Burlingame, CA, USA) was also performed at 4 °C overnight. Immunohistochemical labeling was visualized using a standardized 3,3′diaminobenzidine hydrochloride (10 mg/100 mL phosphate-buffered saline, Sigma-Aldrich, St. Louis, MO, USA) procedure.

### 4.6. Densitometric Measurement

Measurements were performed in the glomerular layers of the left and right OB of the animals. Rats of the non-injected group (n = 3), the Sham + Sham group (n = 5), the 6-OHDA + Sham group (n = 5), and the 6-OHDA + BoNT group (n = 5) were evaluated. Frontal sections of the OB were scanned with a high-resolution scanner (Nexscan F4100, Heidelberger, Germany) using the transmitted light method with a resolution of 2650 dpi. Digital 8-bit grey value images were generated for further computer-aided evaluation. In the images, the glomerular layer was manually delineated using the program Icy 2.0.1.0 (BioImage Analysis unit Institut Pasteur Unite d’analyse d‘images quantitative, Paris, France) to determine the grey values of the glomerular layer and the non-specific background. Data were exported to Excel^®^ and used to calculate the optical density. The negative decadic logarithm of the quotient from the grey values of the glomerular layer and the respective non-specific background resulted in the respective optical densities.

### 4.7. MRI

**Figure 8:**
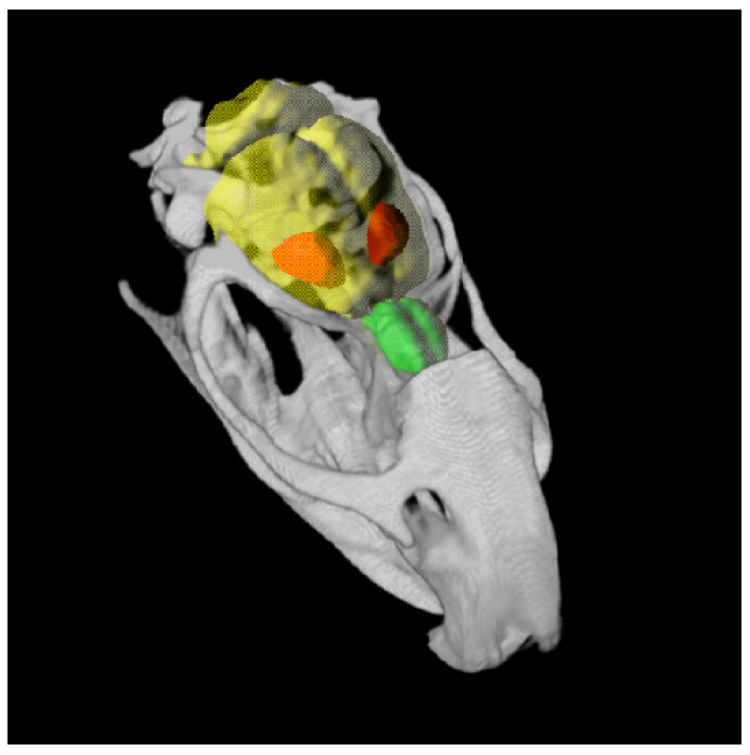
Three-dimensional-rendered computed tomography of a rat skull showing brain (yellow) outlines, including the olfactory bulb (green) and the CPu (red) given by the Schiffer-atlas for topographic orientation.

MRI scans were performed as anatomical references for PET/CT imaging (Figure 8). Each rat received an additional MRI under isoflurane anesthesia vaporized in oxygen gas a few days after the last PET/CT examination. A preclinical MRI scanner (BioSpec 70/30 AVANCE III, 7.0 T, 440 mT/m gradient strength, 7 Tesla, Paravision software v6.01., Bruker BioSpin MRI GmbH, Ettlingen, Germany) with a 1 H transmit resonator (inner diameter: 86 mm; vendor type-nr.: T12053V3, Bruker, Ettlingen, Germany) was used. A receive-only surface coil array (2 × 2 array rat brain coil; vendor type-nr.: T11483V3, Bruker) was placed on the head of the rats during the measurement. Isotropic T1w FLASH imaging sequences with transversal slice orientation, 8/45 ms TE/TR, 35 × 35 × 16 mm FOV, 200 × 200 × 200 µm resolution, 175 × 175 × 80 matrix size in pixels, 20° flip angle, 12:36 min:sec acquisition time; one average and fat suppression were conducted for later anatomical reference, atlas generation, and spatial transformation of the PET/CT images.

### 4.8. Atlas Generation and PET/CT Data Analysis

**Figure 9:**
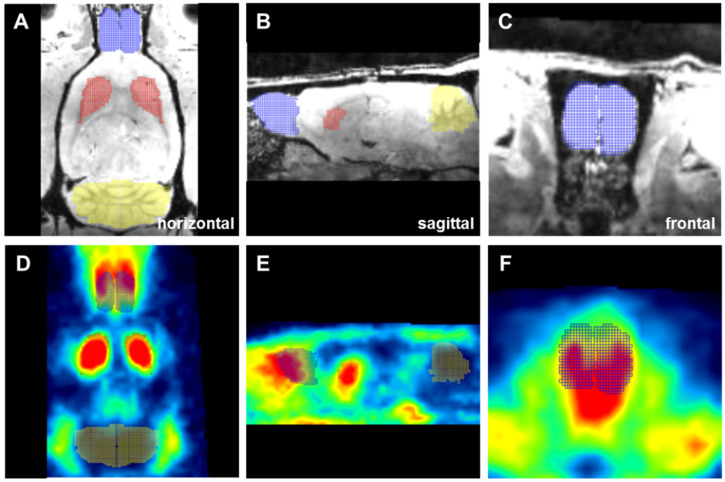
Manual delineation of the OB (blue grid), the cerebellum (yellow grid), and the striatum (red grid) using a group-specific MRI (**A**–**C**) and subsequent definition of VOIs in the PET images with the MRI-derived atlas (**D**–**F**). This figure shows the OB analysis in a representative rat of the Sham + Sham group.

The D_2_/D_3_R availability was quantified with the software PMOD v3.7 (PMOD Technologies LLC, Zurich, Switzerland). A reference model was used, and the non-displaceable Binding Potential (BP_nd_) was determined. For this purpose, the target regions (left and right OB) were delineated in a first step using a self-designed group-specific MRI atlas. For the generation of the atlas, the OB was defined in a representative MRI of the different experimental groups in each slice of the MRI dataset (Figure 9A–C). A 3D-voxel (Schiffer space [158]) fitting the anatomical dimensions of the OB was calculated. The PET/CT data was spatially transformed into the matrix of the Schiffer space via the rat-specific MRI using ridged matching. This protocol guarantees high spatial resolution of the PET/CT data and exact anatomical mapping and was already published [58]. Finally, the generated atlas was overlaid with the PET data, and the voxels of interests (VOIS; here: OB) in the PET dataset were defined. The reference region (cerebellum), being devoid of the target receptor, and an internal control region (striatum) were delineated with the MRI-based rat brain Schiffer atlas [158] (Figure 9D–F). Time-activity curves (TACs) representing the changes in radioactivity concentration over time were extracted. These TACs were used for kinetic modeling with the PKIN tool. The BP_nd_ was calculated using the Simplified Reference Tissue Model 2 (SRTM2).

### 4.9. Tract-Tracing-Based Generation of Connectome Data

To verify whether neuronal connections between the CPu and the main olfactory bulb (MOB) in the 6-OHDA lesion model and BoNT-A therapy approach could possibly be responsible for the improvement in smelling capability, a connectome analysis was performed. The basis for the rat nervous system connectome data is a complete meta-study of all tract-tracing original research publications imported into the neuroVIISAS framework [159]. The directional and weighted data were validated [160]. For connectomics, the MOB was analyzed to differentiate clearly from the connections of the accessory olfactory bulb. Since the term OB is usually used in the literature, instead of MOB, we also use OB here.

### 4.10. Statistical Analysis 

In general, an overall significance level *p* = 0.05 was used. Normally distributed data were subjected to one-way ANOVA or two-way ANOVA using SigmaPlot 14 Software (Systat Software, Inc., San Jose, CA 95110, USA). In the case of statistically significant different mean values, data were subjected to all pairwise multiple comparison procedures (Holm–Sidak method).

If the normality test (Shapiro–Wilk) or equal variance test (Brown–Forsythe) failed, a Kruskal–Wallis one-way ANOVA was done on ranks. In the case of statistically significant different median values among the treatment groups, a multiple comparison procedure (Dunn’s Method) was used.

To determine the strength of association of each behavioral test to apomorphine- or amphetamine-induced rotations, we performed correlation analyses. Prior to correlation, the data were subjected to a Shapiro-Wilk normality test. In the case of normally distributed data, the Pearson product moment correlation test was done. The Pearson product moment correlation coefficient is a parametric test that does not require the variables to be assigned as independent and dependent. Instead, only the strength of association is measured. If the Shapiro–Wilk normality test failed (no normal distribution), Spearman rank-order correlation was performed. Spearman rank-order correlation is a nonparametric test that does not require the data points to be linearly related with a normal distribution about the regression line with constant variance. The Spearman rank-order correlation coefficient does not require the variables to be assigned as independent and dependent. Instead, only the strength of association is measured. Regression lines and prediction intervals were inserted into the resulting scatter plots. Prediction intervals, also called the confidence interval for the population, describe the range where the data values will fall a percentage of the time for repeated measurements.

## 5. Conclusions

We present here a longitudinal study of changes in D_2_/D_3_ receptor availability in the olfactory bulb of the 6-OHDA-induced hemi-PD rat model and results for buried pellets tests. Because olfactory performance appears to be unchanged in hemi-PD rats, our results suggest that the 6-OHDA model may not be appropriate to study olfactory capacity in the context of dopaminergic neurodegeneration in the SNpc. Nevertheless, this study provides first insights into the changes in the olfactory system after intrastriatal BoNT-A injection in hemi-PD rats. In these 6-OHDA + BoNT rats, a relative interhemispheric increase in the availability of D_2_/D_3_ receptors in the OB may be induced via indirect connectivity between the CPu and the OB.

## Figures and Tables

**Table 1:** Overview of rat-specific single BP_nd_ values of the D_2_/D_3_R for the left and right OB, as well as the interhemispheric difference relative to the left hemisphere, in [%] in all rats analyzed. Data are shown for three PET/CT scans of the longitudinal study design (PET/CT 1: one month post-BoNT-A or Sham-BoNT-A, PET/CT 2: three months post-BoNT-A or Sham-BoNT-A, PET/CT 3: six months post-BoNT-A or Sham-BoNT-A). n/a indicates that no data were analyzed due to incorrect tracer injection or no data acquisition.

Group	PET/CT 1			PET/CT 2			PET/CT 3		
	BP_nd_ Left	BP_nd_ Right	[%]	BP_nd_ Left	BP_nd_ Right	[%]	BP_nd_ Left	BP_nd_ Right	[%]
Sham + Sham	3.34	3.27	−1.98	2.39	2.53	5.81	n/a	n/a	n/a
Sham + Sham	2.2	2.29	4.11	2.77	2.83	2.08	3	3.16	5.28
Sham + Sham	2.73	2.79	2.03	2.55	2.52	−1.14	n/a	n/a	n/a
Sham + Sham	3.33	3.46	3.87	3.12	3.13	0.24	2.78	2.67	−4.18
Sham + Sham	3.01	2.97	−1.45	3.3	3.17	−3.95	3.42	3.35	−2.2
Sham + Sham	n/a	n/a	n/a	3.38	3.29	−2.71	3.24	3.26	0.65
Sham + Sham	n/a	n/a	n/a	2.72	2.78	2	3.1	3.07	−0.98
Sham + Sham	n/a	n/a	n/a	3.07	3.1	1.06	3.14	3.28	4.51
Sham + Sham	n/a	n/a	n/a	2.9	3.15	8.89	n/a	n/a	n/a
|Mean| ± SEM	2.93 ± 0.26	2.96 ± 0.29	1.32 ± 1.66	2.91 ± 0.204	2.94 ± 0.21	1.36 ± 1.24	3.11 ± 0.24	3.13 ± 0.26	0.51 ± 1.51
6-OHDA + Sham	2.5	2.44	−2.22	2.61	2.54	−2.68	2.5	2.56	2.15
6-OHDA + Sham	3.11	3.11	−0.02	n/a	n/a	n/a	4.07	3.98	−2.22
6-OHDA + Sham	2.8	2.79	−0.44	2.59	2.69	3.74	3.13	3.04	−3.05
6-OHDA + Sham	2.93	3.08	5.3	3.22	3.42	6.24	3.66	3.85	5.33
6-OHDA + Sham	2.06	2.06	0.37	n/a	n/a	n/a	n/a	n/a	n/a
6-OHDA + Sham	3.45	3.73	8.31	n/a	n/a	n/a	n/a	n/a	n/a
6-OHDA + Sham	n/a	n/a	n/a	2.4	2.56	6.67	3.43	3.53	2.8
6-OHDA + Sham	2.61	2.65	1.48	2.25	2.28	0.98	3.89	3.85	−1.01
6-OHDA + Sham	n/a	n/a	n/a	2.9	2.82	−2.8	3.36	3.39	0.9
|Mean| ± SEM	2.78 ± 0.22	2.84 ± 0.24	1.83 ± 1.40	2.67 ± 0.24	2.72 ± 0.26	2.03 ± 1.51	3.44 ± 0.22	3.46 ± 0.24	0.70 ± 1.40
6-OHDA+BoNT	2.2	2.45	11.62	n/a	n/a	n/a	2.21	2.49	12.66
6-OHDA+BoNT	1.86	1.88	0.88	3.02	3.17	4.87	1.32	1.39	5.12
6-OHDA+BoNT	3.33	3.66	10	3.2	3.44	7.7	n/a	n/a	n/a
6-OHDA+BoNT	2.22	2.52	13.67	3.16	3.2	1.54	3.34	3.56	6.75
6-OHDA+BoNT	1.87	2.11	12.84	n/a	n/a	n/a	2.79	3.07	10.18
6-OHDA+BoNT	3.58	3.81	6.26	3.07	3.32	8	3.6	3.74	4.01
6-OHDA+BoNT	2.73	2.76	1.42	2.77	2.96	6.94	2.86	3.14	9.65
6-OHDA+BoNT	3.61	3.91	8.25	3.69	4	8.49	4.58	4.97	8.44
6-OHDA+BoNT	3.24	3.63	11.96	3.44	3.74	8.68	4.4	4.89	11.28
6-OHDA+BoNT	n/a	n/a	n/a	2.68	2.99	11.71	4.22	4.75	12.71
|Mean| ± SEM	2.74 ± 0.20	2.98 ± 0.21	8.54 ± 1.24	3.13 ± 0.21	3.36 ± 0.23	7.24 ± 1.31	3.26 ± 0.20	3.56 ± 0.21	8.98 ± 1.24

## Data Availability

Not applicable.

## Supplementary Materials

The following are available online at https://www.mdpi.com/article/10.3390/toxins14020094/s1, Figure S1: The latencies to find the pellet in the buried pellet test are correlated with the optical densities of the glomerular layers of the left OB of rats of the 6-OHDA + Sham and Sham + Sham groups (A), and the 6-OHDA + BoNT and 6-OHDA + Sham groups (C), and with the respective optical densities of the glomerular layers of the right OB of rats of the 6-OHDA + Sham and Sham + Sham groups (B), and the 6-OHDA + BoNT and 6-OHDA + Sham groups (D). Neither parameters showed significant correlations. Regression lines are displayed as solid lines and prediction intervals as dashed lines.; Figure S2: The optical densities of glomerular layers of the left OB are correlated with apomorphine-induced rotations of rats of the 6-OHDA + Sham and Sham + Sham groups (A), and 6-OHDA + BoNT and 6-OHDA + Sham groups (B). Optical densities are correlated with the respective amphetamine-induced rotations of rats of the 6-OHDA + Sham and Sham + Sham groups (C), and the 6-OHDA + BoNT and 6-OHDA + Sham groups (D). No significant correlations between these parameters were found. Regression lines are displayed as solid lines and prediction intervals as dashed lines.; Figure S3: The optical densities of the glomerular layers of the right OB are correlated with apomorphine-induced rotations of rats of the 6-OHDA + Sham and Sham + Sham groups (A), and the 6-OHDA + BoNT and 6-OHDA + Sham groups (B), and with the respective amphetamine-induced rotations of rats of the 6-OHDA + Sham and Sham + Sham groups (C), and the 6-OHDA + BoNT and 6-OHDA + Sham groups (D). No significant correlations between these parameters were found. Regression lines are displayed as solid lines and prediction intervals as dashed lines.

## Abbreviations

BoNT-A	Botulinum neurotoxin-A
BP_nd_	non-displaceable Binding Potential
BSA	bovine serum albumin
CPu	striatum (caudate-putamen)
CT	computed tomography
GABA	gamma-aminobutyric acid
hemi-PD	hemiparkinsonian
D_2_/D_3_R	D_2_/D_3_ receptor
MFB	medial forebrain bundle
MOB	main olfactory bulb
MRI	magnetic resonance imaging
OB	olfactory bulb
OBG	olfactory-basal ganglia-connectivity
6-OHDA	6-hydroxydopamine
PBS	phosphate-buffered saline
PD	Parkinson’s disease
PET	Positron emission tomography
PVE	partial volume effect
SNpc	substantia nigra pars compacta
SRTM2	Simplified Reference Tissue Model 2
TAC	time-active hydroxylase
TH	tyrosine hydroxylase
VOI	voxels of interest
VTA	ventral tegmental area
