# Supplementary material for: Olfactory Bulb D2/D3 Receptor Availability after Intrastriatal Botulinum Neurotoxin-A Injection in a Unilateral 6-OHDA Rat Model of Parkinson’s Disease
Source: Toxins (Basel). 2022 Jan 25;14(2):94. doi: 10.3390/toxins14020094 (PMC8879205; doi:10.3390/toxins14020094)
Supplement: Supplementary file 1 [file toxins-14-00094-s001.zip › toxins-1519528-supplementary.pdf]

Supplementary Materials

Olfactory Bulb D_2/D_3 Receptor Availability after Intrastriatal Botulinum Neurotoxin—A Injection in a Unilateral 6-OHDA Rat Model of Parkinson's Disease

Teresa Alberts, Veronica Antipova, Carsten Holzmann, Alexander Hawlitschka, Oliver Schmitt, Jens Kurth, Jan Stenzel, Tobias Lindner, Bernd J. Krause, Andreas Wree and Martin Witt

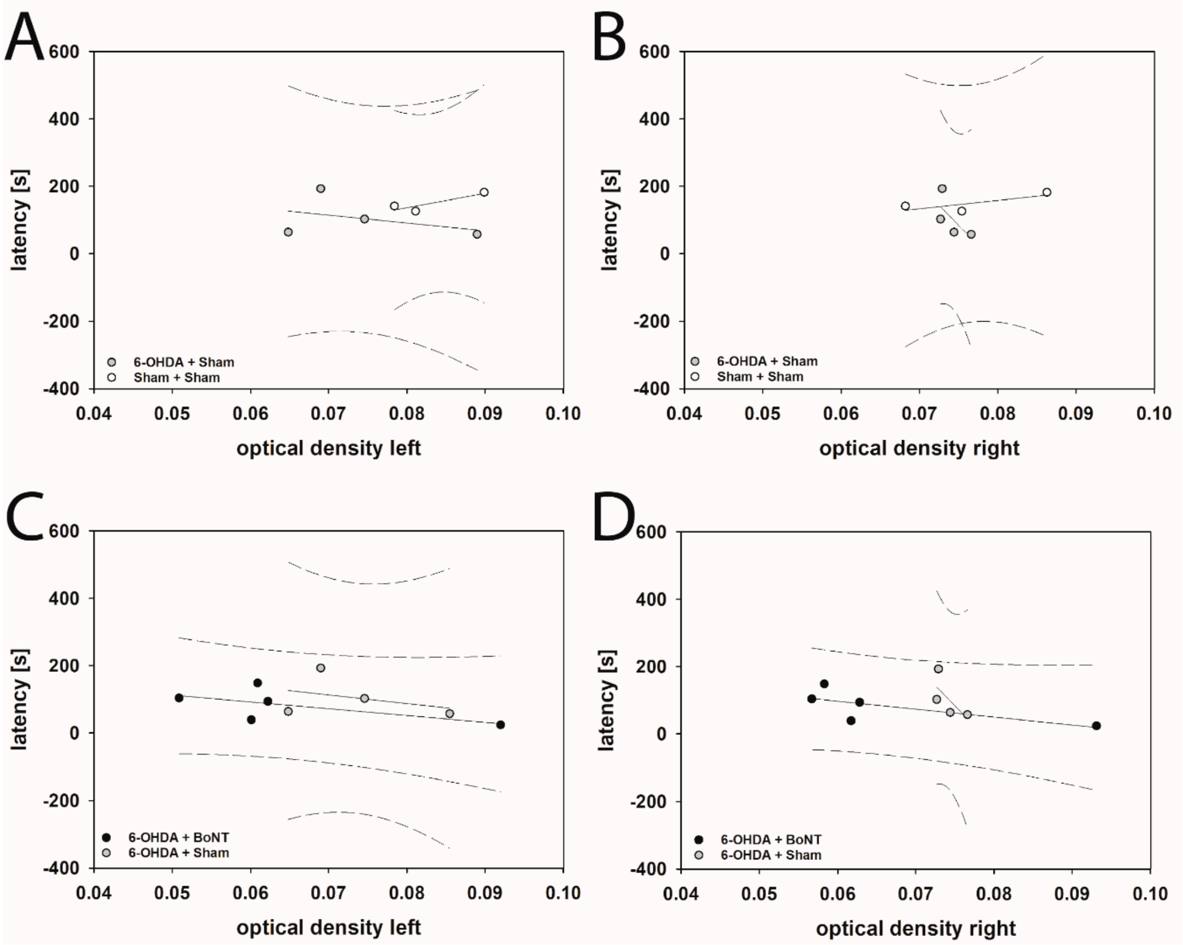

Figure S1. The latencies to find the pellet in the buried pellet test are correlated with the optical densities of the glomerular layers of the left OB of rats of the 6-OHDA + Sham and Sham + Sham groups (**A**), and the 6-OHDA + BoNT and 6-OHDA + Sham groups (**C**), and with the respective optical densities of the glomerular layers of the right OB of rats of the 6-OHDA + Sham and Sham + Sham groups (**B**), and the 6-OHDA + BoNT and 6-OHDA + Sham groups (**D**). Neither parameters showed significant correlations. Regression lines are displayed as solid lines and prediction intervals as dashed lines.

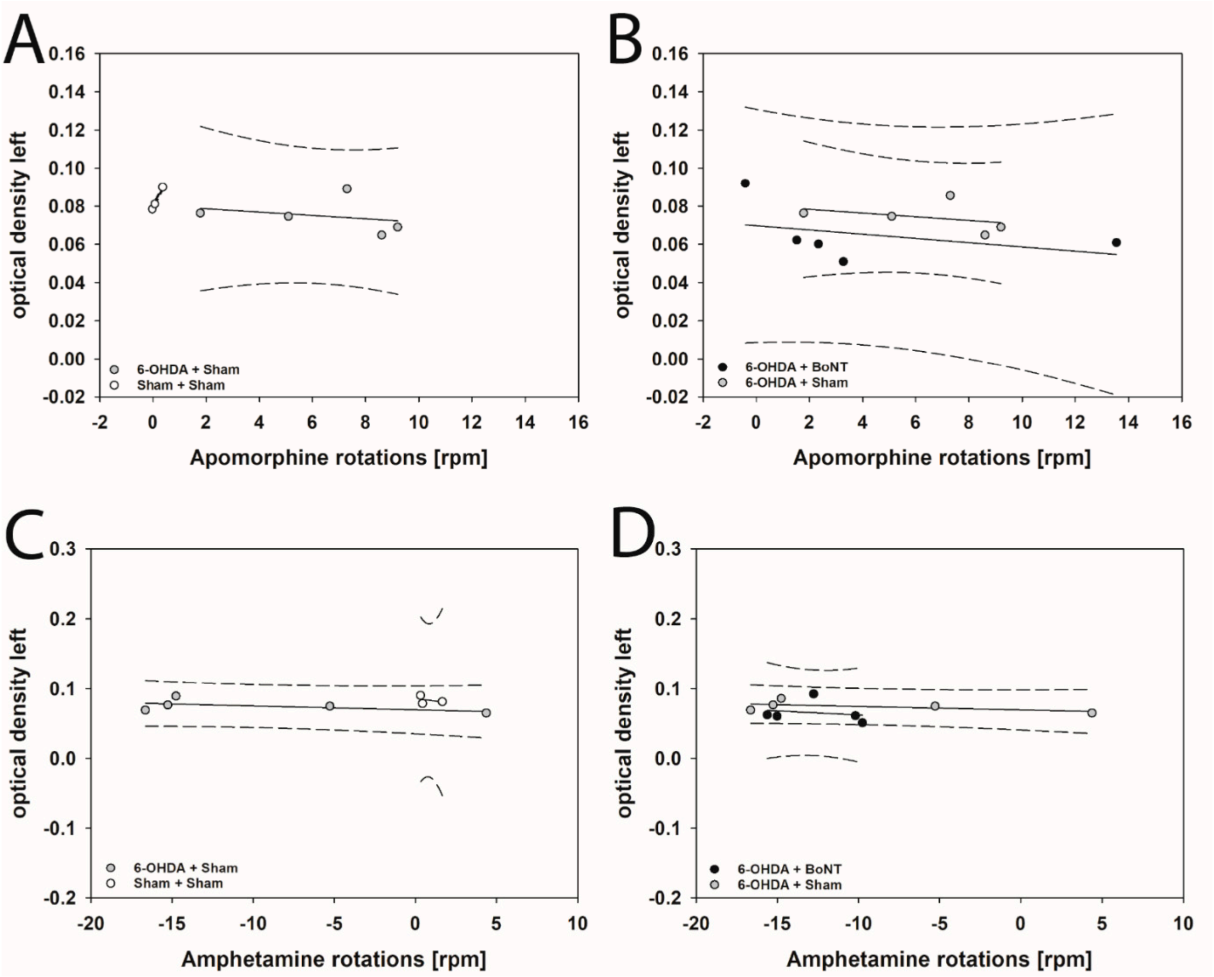

Figure S2. The optical densities of glomerular layers of the left OB are correlated with apomorphine-induced rotations of rats of the 6-OHDA + Sham and Sham + Sham groups (**A**), and 6-OHDA + BoNT and 6-OHDA + Sham groups (**B**). Optical densities are correlated with the respective amphetamine-induced rotations of rats of the 6-OHDA + Sham and Sham + Sham groups (**C**), and the 6-OHDA + BoNT and 6-OHDA + Sham groups (**D**). No significant correlations between these parameters were found. Regression lines are displayed as solid lines and prediction intervals as dashed lines.

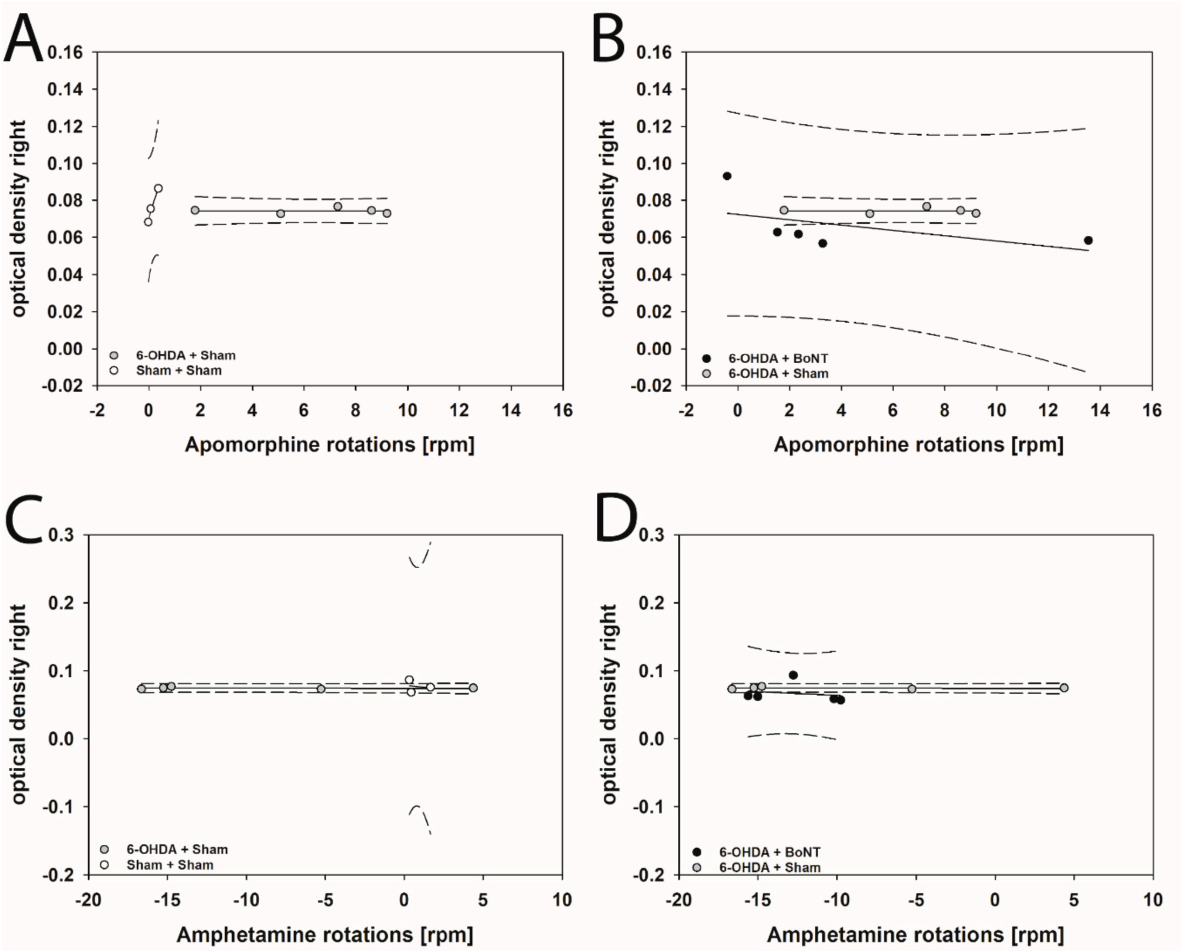

Figure S3. The optical densities of the glomerular layers of the right OB are correlated with apomorphine-induced rotations of rats of the 6-OHDA + Sham and Sham + Sham groups (**A**), and the 6-OHDA + BoNT and 6-OHDA + Sham groups (**B**), and with the respective amphetamine-induced rotations of rats of the 6-OHDA + Sham and Sham + Sham groups (**C**), and the 6-OHDA + BoNT and 6-OHDA + Sham groups (**D**). No significant correlations between these parameters were found. Regression lines are displayed as solid lines and prediction intervals as dashed lines.
